# Afamelanotide Is Associated with Dose-Dependent Protective Effect from Liver Damage Related to Erythropoietic Protoporphyria

**DOI:** 10.3390/life13041066

**Published:** 2023-04-21

**Authors:** Anna-Elisabeth Minder, Xiaoye Schneider-Yin, Henryk Zulewski, Christoph E. Minder, Elisabeth I. Minder

**Affiliations:** 1 Division of Endocrinology, Diabetology, Porphyria and Clinical Nutrition, Stadtspital Zürich, Triemli, 8063 Zurich, Switzerland; 2 Swiss Reference Centre for Porphyrias, Stadtspital Zürich, Triemli, 8063 Zurich, Switzerland; 3 Institute of Laboratory Medicine, Stadtspital Zürich, Triemli, 8063 Zurich, Switzerland; 4 Faculty of Medicine, University of Zurich, 8006 Zurich, Switzerland; 5 Department of Biosystems Science and Engineering (D-BSSE), ETH, 8092 Zurich, Switzerland; 6 Faculty of Medicine, University of Basel, 4001 Basel, Switzerland; 7 Department of Social and Preventive Medicine, University of Bern, 3012 Bern, Switzerland

**Keywords:** Erythropoietic protoporphyria, liver damage, afamelanotide, dose effect

## Abstract

In animal models, melanocyte-stimulating hormones (MSHs) protect the liver from various injuries. Erythropoietic protoporphyria (EPP), a metabolic disorder, leads to the accumulation of protoporphyrin (PPIX). In addition to the most prominent symptom of incapacitating phototoxic skin reactions, 20% of EPP patients exhibit disturbed liver functioning and 4% experience terminal liver failure caused by the hepatobiliary elimination of excess PPIX. Skin symptoms are mitigated through the application of the controlled-release implant afamelanotide, an α-MSH analog, every sixty days. Recently, we showed that liver function tests (LFTs) improved during afamelanotide treatment when compared to before treatment. The present study investigated whether this effect is dose-dependent, as the evidence of dose dependency would support a beneficial influence of afamelanotide. Methods: In this retrospective observational study, we included 2933 liver-function tests, 1186 PPIX concentrations and 1659 afamelanotide implant applications in 70 EPP patients. We investigated whether the number of days since the preceding afamelanotide dose or the number of doses during the preceding 365 days had an effect on LFTs and PPIX levels. In addition, we assessed the effect of global radiation. Results: Inter-patient differences exerted the most prominent effect on PPIX and LFTs. In addition, PPIX increased significantly with an increase in the number of days since the last afamelanotide implant (*p* < 0.0001). ALAT and bilirubin decreased significantly with an increasing number of afamelanotide doses in the preceding 365 days (*p* = 0.012, *p* = 0.0299, respectively). Global radiation only influenced PPIX (*p* = 0.0113). Conclusions: These findings suggest that afamelanotide ameliorates both PPIX concentrations and LFTs in EPP in a dose-dependent manner.

## 1. Introduction

Melanocyte-stimulating hormones (MSHs)—consisting of the three endogenous peptides, α-, β-, and γ-MSH, and their analogs, which are primarily known for inducing skin-pigmentation—have been demonstrated to protect the liver from a broad spectrum of injuries in animal models and cell culture studies [1]. These liver injury models cover a wide diversity of pathophysiological conditions, including acetaminophen-, tetrachloride-, thioacetamide-, and LPS-toxicity, as well as bile-duct ligation, hepatectomy, and acute phase reactions.

These above-mentioned liver-protecting effects are likely based on the anti-inflammatory actions of the MSHs, which they elicit by binding to and activating specific receptors, which are referred to as melanocortin receptors (MCRs) and have five known subtypes (MC1R to MC5R). Signaling from different MCR subtypes similarly contributes to these effects depending on the context, such as in different physiological or pathological conditions, differing tissues, or peptide concentrations [1].

The MSHs apparently mediate these remarkable defensive effects by preventing the decay of the inhibitor (IκB) of nuclear factor-kappa-B (NF-κB), which keeps the inactive IκB/NF-κB complex intact [2,3]. This prevents the translocation of NF-κB into the cell nucleus, where this essential nuclear factor activates the transcription of many molecules involved in the inflammatory process.

Indeed, in the above-mentioned liver injury models, α-MSH prevented the NF-κB activation, the upregulation of the inducible nitric oxidase, and the upregulation of the tumor necrosis factor alpha [2,3]. In the liver damage model induced by carbon tetrachloride, it attenuated TGF-beta1-, collagen alpha1-, and cell adhesion molecule mRNA expression; upregulated matrix metalloproteinase activity and even reversed the established liver fibrosis [4].

Although the above-mentioned data from animal studies, indicating the potential therapeutic effectiveness of the MSHs on a variety of liver diseases, have been known for years, to our knowledge, no study addressing this potential in humans has been performed so far—apart from our recently published retrospective study in erythropoietic protoporphyria (EPP) [5]. This may, in part, be due to the specific pharmacological characteristics of MSHs. As peptides, they must be administered parenterally. Moreover, in vivo, their half-life is only a few minutes, which warrants specific formulations [6]. The α-MSH analog, afamelanotide, a tridecapeptide differing from the endogenous hormone α-MSH by only two positions (4 and 7), has a prolonged half-life compared to the endogenous hormone. It is available as a controlled-release implant administered to prevent phototoxic skin reactions in EPP [7,8]. Afamelanotide, similar to the naturally occurring α-MSH, binds to four of the five MCRs—namely, MC1R, MC3R, MC4R, and MC5R—whereby the binding affinity, at least to MC1R, is higher than that of α-MSH.

EPP is an ultra-rare inherited disorder of heme biosynthesis, with a prevalence of about 1:100,000 [9,10,11]. In most patients, the last enzyme of heme biosynthesis, ferrochelatase, is decreased by more than 70% due to the combination of a deleterious mutation on one allele and a low-expressed second allele caused by the polymorphism c.315-48 T > C (OMIM 177′000). In the minority of patients, an activating mutation of the rate-limiting enzyme of this pathway, aminolevulinic acid synthase 2 (ALAS2), is present (OMIM 300752) or a destructive mutation is present in the CPLX gene [12,13]. All conditions lead to an accumulation of excess protoporphyrin IX (PPIX), one of the substrates of ferrochelatase in the maturating erythroblasts, during the late stages of erythropoiesis. This excess PPIX, a strongly hydrophobic compound, is carried with the reticulocytes from the bone marrow into the bloodstream, where it diffuses out of the erythrocytes and binds to albumin. Because of its hydrophobicity, the major elimination pathway of excess PPIX is the hepatobiliary route. This biliary burden of PPIX mainly causes cholestatic damage to the hepatobiliary system [14,15]. Up to 20% of EPP patients show abnormal liver function tests (LFTs), and terminal liver failure occurs in about 4% [16,17]. Until now, no protective measures to avoid the progression of liver damage to liver failure have been proven to be effective, although a number of interventions have shown a short-term benefit in singular cases [18,19]. As the natural course of EPP-related liver disease has not been studied in a larger group of patients, we cannot exclude that some fluctuations between progression and improvements in the liver condition exist. Consequently, the assumed positive effects described for such interventions in single cases could also reflect the natural course of the disease, or they may be due to the elimination of concomitant damaging influences, such as alcohol consumption or other liver toxins, during the medical management of liver deterioration. Indeed, none of those interventions have prevented death or liver transplantation in the long term in those cases [18].

The excess PPIX circulating in the blood capillaries of the skin of EPP patients is activated by irradiating light in the visible range. Thus, light exposure of the skin leads to the most prominent symptoms of EPP—the incapacitating phototoxic skin reactions—through the formation of oxygen radicals and the damage caused by them. For the prevention of such phototoxic reactions, we have been treating EPP patients with afamelanotide since 2006, when the first trial of afamelanotide in humans was started [7]. Afamelanotide induces skin pigmentation and has anti-inflammatory as well as anti-oxidative properties [20]. These effects improve light tolerance in EPP, as proven by multiple clinical and observational trials and studies [7,8,21,22,23,24,25]. We recently published the positive effect of afamelanotide on LFTs in humans for the first time in a retrospective analysis of the safety data of our EPP cohort treated with afamelanotide [5], with patients who had been observed since 1993 and treated with afamelanotide since 2006. We found a significant decrease in aspartate transaminase (ASAT) and PPIX during afamelanotide treatment. Based on one of the liver damage models, we assumed this effect is more likely due to MC4R rather than MC1R activation [26].

However, due to the study’s retrospective nature, we cannot exclude other potentially improving factors during the afamelanotide application with certainty. Particularly, PPIX concentrations could be influenced by the fact that patients exposed to sunlight for longer periods during afamelanotide treatment compared to before. Based on in vitro tests, it is assumed that the light irradiating into the skin may destroy some of the excess PPIX, hence reducing its levels in the blood. This, in turn, may improve liver function, as less PPIX needs to be cleared from the blood and excreted into the bile by the liver, thus reducing the toxic effects of PPIX on the biliary system. However, the effectiveness of light irradiation on the reduction in PPIX blood concentrations in EPP patients has never been proven.

In the current study, for the first time, we demonstrate that the effects of afamelanotide on liver function and PPIX concentrations are dose-dependent, and we can separate those from the effect of light exposure on PPIX concentrations in an enlarged cohort of EPP patients. This finding suggests a specific protective effect of afamelanotide on EPP-related liver dysfunction.

## 2. Patients, Material, and Methods

### 2.1. Patients, Treatment Schedules, and Safety Measurements

Seventy adult EPP patients, both Swiss and non-Swiss residents, treated at our outpatient clinic since 1993 were included, comprising 16,509 laboratory data with 4119 values related to PPIX and liver function, and 1659 afamelanotide implant administrations. The age of the patients at the study’s end (31 December 2020) ranged between 20 and 81 years (mean ± SD = 43 ± 16, median 41).

During the first clinical trial (CUV010) two implants, each containing 20 mg of afamelanotide, were administered to each of the five study participants at a sixty-day interval [27]. The formulation of the implants differed from the later-used ones, but also was a slow-release formulation. Thereafter, all used implants contained 16 mg of afamelanotide (SCENESSE^®,^ Clinuvel Pharmaceuticals, Melbourne, Australia). The CUV017 was a cross-over study. Therefore, each study participant received three doses of the active substance and three doses of a placebo, so the interval between the two active doses was 120 days. During the compassionate use program from the years 2008 to 2012, the yearly administered number was 5–6 implants, as required for optimal protection from phototoxic reactions [22]. They were usually administered at a sixty-day interval, with some winter pauses in less-affected patients. In the year 2016, reimbursement difficulties caused an interruption of the treatment in some patients. Thereafter, the number of administered implants per year was influenced, in part, by the negotiations with the insurers. Later, for some patients, the numbers were gradually adapted to meet their medical needs.

The local ethics committee approved the study (BASEC-No. 2018-00131, 2018-00758), and all patients gave their written informed consent prior to the start of the study. One patient, for whom all patient data have been included in the study, intermittently had a strong increase in his liver enzymes due to a histologically verified allergic drug reaction of the liver to acetaminophen, which subsided after its discontinuation. Thirty-eight patients were identical to those included in our previous study [5]. However, we included additional data from these patients, as we extended the data collection time up to the end of 2020. Further, we could add another 32 patients to this study, as, in contrast to our prior investigation, no pretreatment data were required for the current study.

Safety laboratory tests were performed approximately every six months under treatment, with additional data collected in special circumstances e.g., prior to treatment, for diagnosis, or for the follow-up of EPP, in order to assess the risk of EPP-related liver disease or other clinical reasoning for more intense laboratory examinations. In addition, data from the CUV010 and CUV017 clinical trials, as well as from the early compassionate use program were included, which also involved more intense safety testing.

### 2.2. Data Sources and Statistical Analysis

All available anonymized data from the patients were included, which also comprised those available before the first dose of afamelanotide. Data were collected from our electronic laboratory system, from patient documents, and provided by CLINUVEL (CUV010 and CUV017 clinical trials). The data were processed in R [28]. We eliminated duplicates coming from different sources. Only laboratory tests that had been performed in sufficient numbers (i.e., >250 data per test) were included in the analyses. This limited our analysis to the following LFTs: Bilirubin, aspartate transaminase (ASAT), alanine transaminase (ALAT), and gamma-glutamyltransferase (GGT). For each blood collection date, the number of days since the last dose (truncated at 730 days) and the number of implants administered in the 365 or 730 preceding days were calculated, using Excel Version 2016.

The monthly global radiation intensity data of 60 locations distributed over the whole of Switzerland were averaged and then standardized to the mean of those measurements of global radiation denominated as relative global radiation. One unit of this standardized relative global radiation corresponds to 137.1 W/m^2^. The standardized monthly values were calculated according to the month of the blood collection or implant application date, respectively.

### 2.3. Assessment of Dose-Dependent Effects of Afamelanotide

Currently, afamelanotide is applied as a 16 mg controlled-release implant approximately every sixty days. Therefore, a maximum of six to seven doses are administered within one year (365 days). In the countries of the European Union, a maximum of four doses per year was recommended by the European medicine agency (EMA), whereby the number of yearly doses was considered to be a clinical decision to be made by the treating physician [29]. In contrast, both the FDA (USA) and TGA (Australia) regulatory agencies recommended an application every sixty days without limitations on the number of implants per year. There is no evidence supporting an increased number or more severe adverse events with more yearly afamelanotide doses [22]. In Switzerland, the number of implants per year, which are reimbursed by insurers, is negotiated individually for every patient. Many patients required 5–6 doses per year, as they were symptomatic throughout the year, being either sensitive to artificial light, living in areas with a lot of snow and sunshine in winter as provocative factors against severe phototoxicity, or performing professional and/or social activities exposing them to harmful light throughout the year.

We defined two indices as indicators for a potential dose effect of afamelanotide on specific laboratory tests.

First, we aimed to assess whether there is an **immediate dose response (IDR)** to afamelanotide. As a basis, we determined, for each blood test, the number of days since the last implant (index_IDR_). Delays of more than 729 days were truncated at 730 days (i.e., more than 2 years), with the assumption that the long-term effects of afamelanotide would have fully faded within this time. If the patient had never had an implant before the blood draw, an identical interspace of 730 days was used.

Secondly, we aimed to assess the **cumulative dose effect (CDE)**. For this, we calculated the number of implants the patient had received during the preceding 365 or 730 days of an implant, respectively (index_CDE365_ or index_CDE730_).

For both indices, implants administered on the same date as the blood tests were not counted.

These two indices of dose effects are not independent of each other, as a patient receiving six implants per year likely has an interval since the last dose of afamelanotide of about 60 days. However, a patient with three implants per year may have variable intervals, namely all between 2 and 8 months.

### 2.4. Further Statistical Analyses

Further statistical analyses were conducted in Analyse-it for Excel, Version 4.51. In case the effect of more than one independent variable on a dependent variable was tested, we used multiple linear regression analysis. For the dose effects of repeated administration over 730 days, we expected them to gradually level off, especially as they could progressively approach the reference values, as evident in our previous study [5]. Therefore, we assumed a non-linear correlation between a dependent and independent variable and used exponential regression analysis for those analyses. In pairwise comparisons, Pearson’s r, Spearman’s rs, or Kendall’s tau were calculated and Spearman’s rs was used to assess significance. A two-sided *p*-value of <0.05 was considered significant.

## 3. Results

### 3.1. General Correlations between LFTs and PPIX

**Figure 1:**
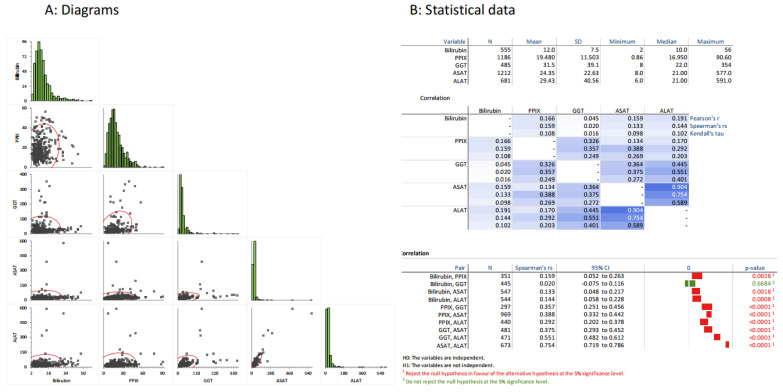
Correlation of PPIX and liver parameters. Diagram (**A**): Histograms (green) show the distribution of the laboratory data. The pairwise comparisons for all laboratory data are displayed. Diagram (**B**): The statistical data for each test, including mean, SD, median, minimum, and maximum, are displayed. Below, pairwise correlations, calculated using three different methods (Pearson’s r, Spearman’s rs, and Kendall’s tau) are provided, whereby the intensity of the underlying blue color indicates the grade of correlation. The lowest part displays the 95% confidence interval and the *p*-values for those pairwise comparisons using Spearman’s rank correlation test.

We first investigated the pairwise correlations of LFTs and PPIX (Figure 1). Here, all LFTs significantly correlated with each other, except for bilirubin with GGT (Figure 1B). The two transaminases correlated the best, as expected. The correlations were always positive, indicating that higher values in one test corresponded to higher values in the other test, as displayed by the diagrams in Figure 1A. Further, all LFTs correlated with PPIX, indicating that liver function influences the PPIX concentration, or vice versa, or that they are influenced by a factor common to both.

The expected correlations found in our dataset confirmed that the data collection resulted in reasonable and, therefore, most likely reliable data.

### 3.2. Distributions of the Index_idr_ and Index_cde_

**Figure 2:**
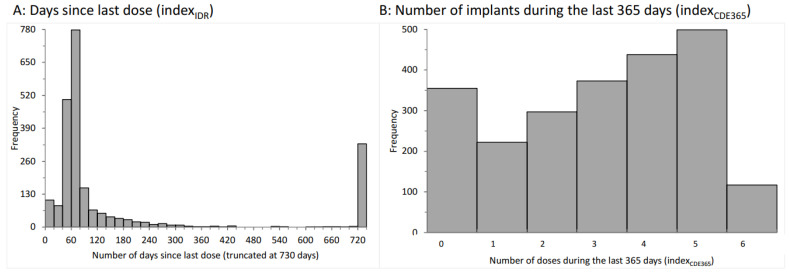
Characteristics of implant applications. Diagram (**A**): A frequency histogram of the interval between blood drawings and days since the last implant dose of afamelanotide is displayed. The data have been truncated at day 730. The same 730-day interval was assumed if the lab tests were performed before any prior afamelanotide dose. Diagram (**B**): a frequency histogram of the number of doses applied during the preceding 365 days is shown.

As indicated above, we evaluated the number of days between the blood tests and the previous implant (Figure 2A). Most intervals centered around 60 days, as expected from the recommendations in the summary of the product characteristics [29]. Additionally, a relatively large number of analyses were performed more than 729 days after the last preceding dose or prior to treatment. The index_IDR_ data served as the basis for IDR assessments.

Second, we determined the number of yearly afamelanotide implants before the blood tests. They ranged from zero to six implants in the preceding 365 days (0 doses: 15%; 1 dose: 10%; 2 doses: 13%; 3 doses: 16%; 4 doses: 19%; 5 doses: 22%; and 6 doses: 5%; Figure 2B) and from zero to 12 in the preceding 730 days (0 doses: 14.2%; 1 dose: 8.0%; 2 doses: 8.3%; 3 doses: 6.0%; 4 doses: 6.1%; 5 doses: 5.9%; 6 doses: 6.4%; 7 doses: 8.2%; 8 doses: 7.9%; 9 doses: 9.3%; 10 doses: 10.4%; 11 doses: 7.7%; and 12 doses: 1.6%). These data served as the basis for CDE360 or CDE730 assessments, respectively.

**Figure 3:**
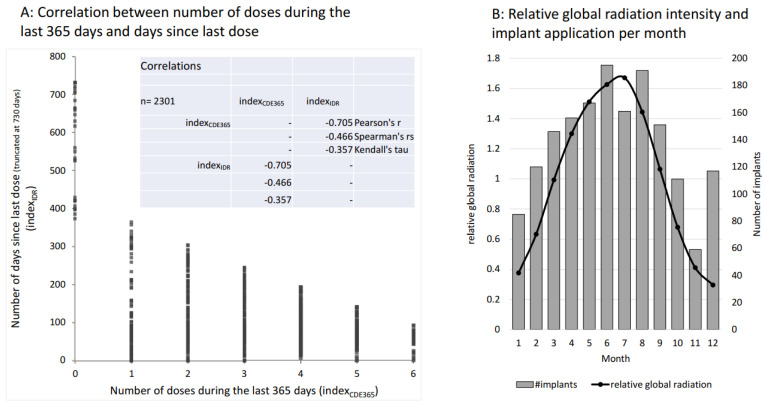
Characteristics of implant applications and global radiation. Diagram (**A**): a strong, negative correlation between the number of doses during the preceding 365 days and the number of days since the last dose exists. Diagram (**B**): A histogram of the number of implant doses per month (1 = January, 2 = February, …, 12 = December) is overlaid by the relative global radiation averaged over 60 locations in Switzerland (1 unit corresponds to 137.1 W/m^2^). They closely resemble each other except for a slight reduction in July and an increase in the months before and after because of the summer holidays and an increase in December, due to the winter holidays).

As stated above, these two indices of dose effects are not independent of each other. Indeed, the indices are highly correlated, as illustrated in Figure 3A. Nonetheless, we considered both indices to be of value to our investigations, as the first index covers the recent and more acute effects of afamelanotide (IDR), whereas the second index represents the long-term effects of afamelanotide treatment (CDE).

### 3.3. Global Radiation as an Independent Effector

The symptoms of EPP, the acute phototoxic skin reactions, are light-induced. The variation in the radiation of natural light largely determines how much the patients are affected. This, in turn, defines in which months of the year the patients most require afamelanotide implant administrations. Indeed, the highest number of implants were administered between March and September, and November was clearly the month with the lowest frequency (Figure 3B). Moreover, the monthly number of implants reflected the relative global radiation in Switzerland, apart from a relatively high number of administrations in December, which was likely due to the expected exposure to a combination of snow and sunshine from December to February, as this is one of the strongest triggering factors for phototoxic reactions in EPP.

Aside from causing acute phototoxic burns in EPP, light irradiation of the skin may destroy the excess PPIX circulating in the body’s bloodstream, and, therefore, lower the PPIX concentrations, which in turn may improve liver function. Therefore, we included global radiation as an independent variable in the analyses.

### 3.4. Immediate Dose-Response and Cumulative Dose Effect of Afamelanotide on PPIX Concentrations and LFTs (Table 1)

Next, we investigated whether PPIX or any of the LFTs correlated with the index_IDR_, the index_CDE_, or the relative global radiation. For this purpose, we used multilinear regression analysis. Thereby, we assessed the effect of the four independent variables—the patient ID (which covers the effects of the individual patient), the index_IDR_ (the number of days since the last implant), the index_CDE365_ (the number of implants during the preceding 365 days) and relative global radiation—on the dependent variables, PPIX concentration or LFTs (bilirubin, ALAT, ASAT, or GGT, respectively). For each calculation, we determined the effects of the patient ID and one additional independent variable on one dependent variable.

In Table 1, the *p*-value for the multilinear model as a whole, as well as *p*-values for each of the independent variables, are given. As can be seen, the patient ID always had a strong significant influence on all dependent variables (PPIX, ALAT, ASAT, bilirubin, and GGT), which indicates that the patient-specific laboratory values exhibit much lower variability than those of the whole cohort. Thus, the inclusion of the patient ID reduced the variability in the models.

Each line in the table represents one multilinear regression analysis, whereby the effects of two independent variables on either PPIX or LFTs were analyzed. The model *p*-value shows the statistical result of each of the multilinear regression analyses. The independent variables always included the patient ID to take the effect of the individual patient into account, which, in all analyses, had a paramount influence. As a second independent variable, one of the following variables was assessed: index_IDR_ [immediate dose response, corresponding to the number of days since last dose], index_CDE365_ [cumulative dose response, corresponding to the number of implants applied during the last 365 days], or relative global radiation, whereby the average global radiation in Switzerland is 137.1 W/m^2^ per month.

For the second independent variable, along with the *p*-value, the effect is also displayed. This effect represents the influence of one unit of the second independent variable on the dependent variable (example 1: for the dependent variable PPIX, the effect of index_IDR_ is calculated as follows. One additional day since the last dose results in an increase in PPIX of 0.008376 μmol/L, or PPIX increases by 3.05 μmol/L if 365 days have passed since the last dose (0.008376 × 365 = 3.05); example 2: the effect of index_CDE365_ on the dependent variable, PPIX—one additional dose within the last 365 days results in a decrease in PPIX of 0.8043 μmol/L, or PPIX decreases by 4.83 μmol/L after 6 doses during the last 365 days (−0.8043 × 6 = −4.83) compared to no treatment; or example 3: the effect of relative global irradiation on PPIX—an additional 137.1 W/m^2^ of radiation (which corresponds to about the difference between irradiation in summer versus winter; see Figure 3B) results in a PPIX reduction of 1.059 μmol/L).

In addition to the patient ID, the quantitative effect of a second independent variable on the dependent variables is listed. This effect corresponds to the change induced in the dependent variable (i.e., by one day for the independent variable, index_IDR_; one implant for the independent variable, index_CDE_; or one relative global radiation unit (137.1 W/m^2^) for the variable “relative global radiation”). Thereby, a positive number indicates a positive correlation, i.e., if the independent variable increases, the dependent variable also increases, and vice versa.

The second independent variable, index_IDR_, had a significant positive effect on the PPIX concentrations, indicating an IDR of afamelanotide on PPIX concentrations. The index_CDE_ used as a second independent variable had a significant negative effect on PPIX, ALAT, and bilirubin, indicating a CDE of afamelanotide on these three LFTs. Relative global radiation only had a significant and, as expected, negative effect on PPIX.

Interestingly, index_IDR_ always had a positive effect on the dependent variable (except for bilirubin), indicating an increase in PPIX, ALAT, ASAT, or GGT concentrations with an increasing time interval since the last dose, although this effect was not always statistically significant. In contrast, the effect of the variable index_CDE_ was always negative, indicating a decrease in PPIX, ALAT, ASAT, bilirubin, and GGT with an increasing number of yearly doses, although this effect reached statistical significance only for PPIX, ALAT, and bilirubin.

**Figure 4:**
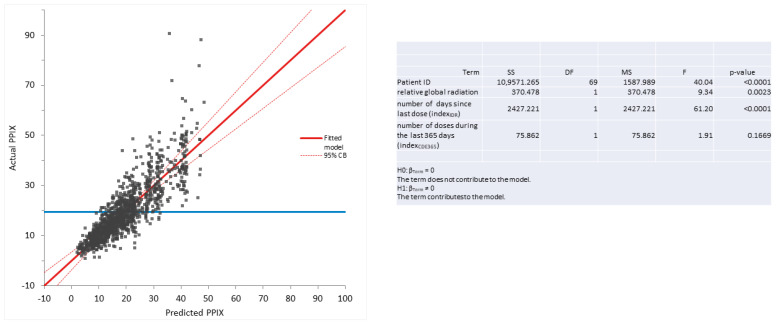
Multilinear regression analysis as a model of prediction for PPIX concentration based on patient ID and the effects of dosing intervals. This diagram illustrates to what extent the applied multivariate statistical model enables the prediction of the PPIX values based on patient ID, the number of doses in the preceding 365 days, and days since the last dose. The correlations and the 95% confidence intervals are displayed in red, and the null model is in blue.

**Figure 5:**
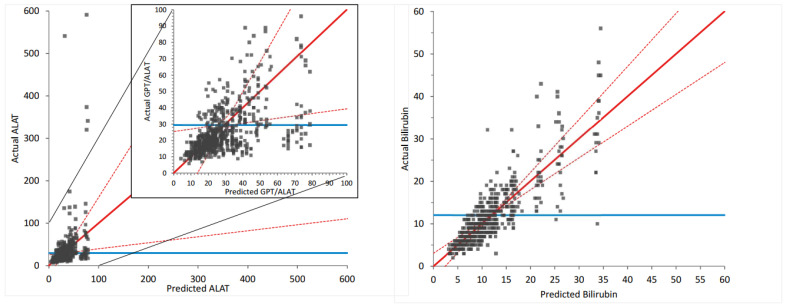
Model of predicted versus actual ALAT (diagram **A**) and bilirubin (diagram **B**). Multilinear regression analysis was used, including patient ID and the number of doses as the two independent variables. For ALAT, the lower part of the diagram was displayed in an enlarged presentation (insert). The correlations and the 95% confidence intervals are displayed in red, and the null model, indicating no correlation between the independent and dependent variables, is in blue.

In an additional multilinear model for PPIX as a dependent variable with patient ID, global irradiation and both index_IDR_ and index_CDE_ as independent variables, besides global irradiartion only index_IDR_ remained significant. PPIX increased by 0.01 μmol/L per day, equaling 6 μmol/L per 2 months. As shown in Figure 4, applying this model allows for the prediction of PPIX values in our patients. These results indicate that PPIX is mainly influenced by the more short-term immediate dose response (IDR), whereas the LFTs are mainly influenced by the long-term cumulative dose effect (CDE). The predicted and actual values also clearly correlate well when assessing ALAT or bilirubin, respectively, as dependent variables and index_CDE_ and patient ID as independent values in multilinear regression (see Figure 5).

**Figure 6:**
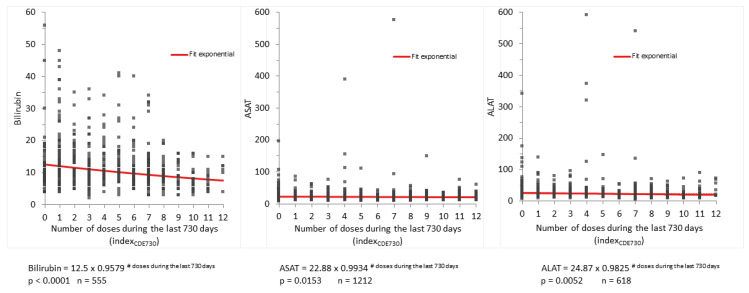
Exponential regression models of bilirubin, ASAT, and ALAT versus the number of doses during the preceding 730 days (two years). The number of doses mostly influenced bilirubin. More than eight doses per two years appear to be required to achieve a significant effect on patient management.

As the number of doses during the preceding 365 days apparently influenced the LFTs positively, we questioned whether this effect is also preserved over a prolonged time span, namely the number of doses during the preceding two years (730 days). We assumed that the effect might be more pronounced initially and level off with time. Therefore, we applied an exponential regression analysis of the number of doses during the preceding 730 days versus the concentrations of the LFTs bilirubin, ASAT and ALAT. They all decreased significantly depending on the number of doses (*p* < 0.0001; *p* = 0.0153, and *p* = 0.0052, respectively). As illustrated by the diagrams in Figure 6, especially the higher values were visibly diminished. From a clinical standpoint, this is most important, as the patients with the highest levels bear the highest liver failure risk. However, those highest test values only diminished if the patients were treated with more than eight doses per two years (i.e., more than four doses per year).

### 3.5. Influence of Global Radiation on PPIX Concentrations and LFTs as Assayed by Multilinear Regression Analysis (Table 1)

Using the same multilinear model as described under Section 3.4, we analyzed the effects of global radiation on PPIX and LFTs, whereby we included patient ID in addition to the relative global radiation as independent variables. These analyses show that increasing global radiation reduced PPIX concentrations significantly but had no significant influence on any of the LFTs. Further, we used an additional multilinear model to assess the effects of both index_IDR_ and the relative global radiation, in addition to patient ID, as independent variables on PPIX concentrations as the dependent variable. All three independent variables had a strong, significant effect on PPIX concentrations (*p* < 0.0001, *p* = 0.0023, and *p* < 0.0001). The effects between the lowest and highest monthly relative global irradiation result in a difference of 1.67 µmol/L PPIX concentrations, whereas the omission of treatment for one year resulted in an increase in PPIX concentrations of 3.1 µmol/L. These results support a photobleaching effect of light exposure on PPIX concentrations in the blood of EPP patients.

## 4. Discussion

In our cohort, PPIX concentrations and LFTs correlated significantly (Figure 1), confirming our previous finding that PPIX concentration is a predictor of EPP-related liver damage. High PPIX concentrations are considered the main risk factor for EPP-related liver damage [16,30,31,32]. Secondarily, a deterioration in liver function can augment PPIX concentrations, as it affects the biliary elimination of excess PPIX. The interrelatedness of both conditions may result in a vicious cycle. Thus, this study supports that, in addition to its protection of the skin from phototoxic reactions, afamelanotide also positively affects both pathophysiological processes—i.e., the production of PPIX and liver damage—as it reduces both PPIX and LFT dose-dependently.

It is unclear whether a healthy liver expresses any melanocortin receptors [33]. However, MC4R is expressed after liver damage. As described by several authors, its activation by an MSH or MSH-analogue leads to favorable effects on these injuries [26,34,35]. The effects are mainly attributed to the inhibition of inflammatory processes, but MSH may also improve regenerative processes and inhibit fibrosis [4]. The anti-inflammatory activity is apparently not only linked to the major pharmacophore HFRW of MSH (corresponding to amino-acids 6–9 of afamelanotide) but, independent of the melanocortin receptors, the terminal tripeptide, KPV, may also be pharmacologically active as an anti-inflammatory agent [36].

In EPP, afamelanotide is administered at a dose of 16 mg every sixty days as a controlled slow-release implant [6]. After administration, it can be detected in the blood circulation for about 7 days, which is less than 12% of the time interval between doses. This pharmacokinetic characteristic results in the minimal and only intermittent exposure of the tissues to afamelanotide, whereby, at least in melanocytes, the intracellular activity of MSH may last longer [37]. Further, the KPV tripeptide may be active at an extremely low concentration [36]. In our study, most blood drawings were completed around sixty days after the preceding implant application (Figure 2A). Despite this, we found significant dose-dependent effects of afamelanotide, with a reduction of PPIX and the LFTs ALAT and bilirubin in our EPP cohort and the strongest association being between the number of doses during the preceding two years (index_CDE730_) and bilirubin (Figure 6). This observation is in line with the hypothesis of a primarily biliary mechanism as an initiator in the evolution of liver damage.

The PPIX concentrations were preponderantly influenced by the interval occurring since the preceding dose, thus indicating a predominant short-term effect of afamelanotide on PPIX concentrations. In contrast, the LFTs diminished in dependence on the number of doses during the preceding 365 or 730 days, indicating a more chronic effect of afamelanotide administration. This correlates to the physiology of the involved tissues. Whereas excess PPIX is produced in the red bone marrow with a rapid turnover of cell production and maturation, liver tissue, in contrast, has a low turnover rate of cell renewal. With respect to the protection of liver function, the three to four doses per year, as imposed as a yearly maximum by the EMA, seem to be insufficient, and at least 9 implants during the previous two years are required (Figure 6). We, therefore, speculate that a shorter time interval of implant applications, such as every four to six weeks, could lead to pronounced positive effects on EPP-related liver damage, for which, currently, there is no effective treatment and which causes liver failure requiring a liver transplant in 2–5% of cases [17].

PPIX concentrations were also reduced with increasing global radiation. However, global radiation had no significant influence on LFTs. This finding makes it unlikely that prolonged sunlight exposure with a concomitant decrease in PPIX is the primary effect of afamelanotide treatment leading, secondarily, to an improvement of liver function. Moreover, the variable time course, a more short-term effect on PPIX concentrations, and a more long-term effect on LFTs support the concept of two independent, beneficial effects of afamelanotide.

Since it has been detected that EPP may lead to terminal liver failure, liver protection has been a focus in its medical management, whereby the avoidance of hepatotoxins, such as alcohol or hepatotoxic drugs, including oral contraceptives, has been promoted [16,18,38]. For the prevention of liver damage in EPP, the application of cholestyramine with the intention to prevent the purported enterohepatic recirculation of PPIX and, thus, reduce its blood levels, has been advocated by McCollough et al. [39]. However, the existence of an enterohepatic recirculation of PPIX is controversial. In fact, a recent study has demonstrated that cholestyramine has no effect on PPIX concentrations in EPP [40].

Another intervention for liver protection in EPP is the application of bile acids with the aim of increasing bile flow and, hence, augmenting the biliary excretion of PPIX [39]. Yet, bile composition is complex and, physiologically, finely adjusted. In an animal model with a genetic EPP, the increase in bile acid content in bile was considered a possible cause for the induction of biliary fibrosis [41]. In the same mouse model, neither ursodesoxycholic acid nor heme arginate improved liver function [42].

In summary, no preventive measures with documented effectiveness for liver damage in EPP exist as of yet. Therefore, most interventions for the treatment of EPP-related liver disease were performed in an advanced stage of liver damage, and the recommendations relied on the medical experiences in single cases. As stated above, the interventions were not compared in a randomized trial or with historic controls, as such controls do not exist. According to our observations, the severity of liver involvement in EPP may fluctuate, as do LFTs. Further, certain proposed interventions, such as hematin infusions or iron supplementation, are not only useless but may even be harmful [43,44].

The dose-dependent effects of afamelanotide on lowering both PPIX and liver enzymes, as demonstrated in this study, favor the application of 5–6 afamelanotide implants per year to prevent EPP-related liver damage, especially in patients at a higher risk for this complication. Patients at a higher risk are those with PPIX concentrations in erythrocytes above 20–30 μmol/L [16,31]. Hence, in the presence of other risk factors, such as liver steatosis [32] or in the case of signs of liver damage, a two-monthly implant application throughout the year should be considered.

Finally, the potential of MSH analogs to treat other liver disorders that are currently without effective treatment is unknown and could be considered and investigated in future studies.

## Figures and Tables

**Table 1:** Multilinear regression analyses of the dose effects of afamelanotide and the relative global radiation on PPIX and LFTs.

Dependent Variable	Model *p*-Value	Independent Variables			
		Patient ID *p*-Value	Second Independent Variable		
			Name	*p* Value	Effect
PPIX	<0.0001	<0.0001	index_IDR_	**<0.0001**	0.008376
PPIX	<0.0001	<0.0001	index_CDE365_	**<0.0001**	−0.8043
PPIX	<0.0001	<0.0001	Relative global radiation	**0.0113**	−1.059
ALAT	0.0008	0.0008	index_IDR_	0.1625	0.009162
ALAT	0.0003	0.0005	index_CDE365_	**0.012**	−2.509
ALAT	0.001	0.0008	Relative global radiation	0.263	−3.6
ASAT	<0.0001	<0.0001	index_IDR_	0.1074	0.004727
ASAT	<0.0001	<0.0001	index_CDE365_	0.1606	−0.5345
ASAT	<0.0001	<0.0001	Relative global radiation	0.4277	−1.086
Bilirubin	<0.0001	<0.0001	index_IDR_	0.9527	−5.90 × 10^−5^
Bilirubin	<0.0001	<0.0001	index_CDE365_	**0.0299**	−0.304
Bilirubin	<0.0001	<0.0001	Relative global radiation	0.5845	0.2239
GGT	<0.0001	<0.0001	index_IDR_	0.2044	0.01082
GGT	<0.0001	<0.0001	index_CDE365_	0.1846	−1.668
GGT	<0.0001	<0.0001	Relative global radiation	0.4821	−2.475

## Data Availability

Data is unavailable due to ethical restrictions.

## Abbreviations

ALAS2	aminolevulinic acid synthase 2
ALAT	alanine transaminase
ASAT	aspartate transaminase
CDE	cumulative dose effect
EMA	European medicine agency
EPP	erythropoietic protoporphyria
FDA	U.S. Food and Drug Administration
GGT	gamma-glutamyltransferase
IDR	immediate dose response
IκB	inhibitor of nuclear factor-kappa-B
LFT	liver function tests
MCR	melanocortin receptor
MSH	melanocyte-stimulating hormone
NF-κB	nuclear factor-kappa-B
PPIX	protoporphyrin
TGA	Therapeutic Goods Administration
